# Deformable and Fragile Object Manipulation: A Review and Prospects

**DOI:** 10.3390/s25175430

**Published:** 2025-09-02

**Authors:** Yicheng Zhu, David Yang, Yangming Lee

**Affiliations:** RoCAL Laboratory, Rochester Institute of Technology, Rochester, NY 14623, USA; yz8733@rit.edu (Y.Z.); freedomdavidyang@gmail.com (D.Y.)

**Keywords:** deformable object manipulation (DOM), fragile object safety, multi-modal sensor fusion, adaptive planning, reflective control, robotics

## Abstract

Deformable object manipulation (DOM) is a primary bottleneck for the real-world application of autonomous robots, requiring advanced frameworks for sensing, perception, modeling, planning, and control. When fragile objects such as soft tissues or fruits are involved, ensuring safety becomes the paramount concern, fundamentally altering the manipulation problem from one of pure trajectory optimization to one of constrained optimization and real-time adaptive control. Existing DOM methodologies, however, often fall short of addressing fragility constraints as a core design feature, leading to significant gaps in real-time adaptiveness and generalization. This review systematically examines individual components in DOM with a focus on their effectiveness in handling fragile objects. We identified key limitations in current approaches and, based on this analysis, discussed a promising framework that utilizes both low-latency reflexive mechanisms and global optimization to dynamically adapt to specific object instances.

## 1. Introduction

Deformable object manipulation (DOM) is a pivotal and complex challenge in robotics, with extensive real-world applications in domains such as robotic surgery, agriculture, food handling, waste sorting, caregiving, and household service [1,2]. Unlike rigid objects, deformable objects exhibit dynamic and variable behaviors that are influenced by their material properties, applied forces, and environmental constraints [3]. These inherent complexities demand advanced strategies for sensing, perception, modeling, planning, and control to enable precise and adaptive manipulation [4].

An overlooked yet critical aspect of DOM is the safe handling of *fragile* deformable objects—those highly susceptible to damage or irreversible deformation due to their structural or material characteristics [5]. This fragility can be categorized as **global**, where cumulative stress affects the entire object (e.g., a glass sheet), or **local**, where damage results from concentrated forces (e.g., bruising a piece of fruit). These objects not only elevate the complexity of manipulation but also impose strict requirements for balancing adaptability with safety [6]. Damage caused by factors such as excessive force, inaccurate modeling, or delayed control responses remains a major challenge for current robotic systems [7,8]. Addressing these safety concerns is of critical importance for expanding the applicability and reliability of autonomous robots.

While deformable object manipulation has been the focus of significant research efforts spanning modeling, perception, learning, and control, the safety-aware handling of fragile deformable objects remains insufficiently explored. Existing review works provide comprehensive overviews of technical advances in DOM, yet few systematically examine the unique challenges of ensuring safety during manipulation of fragile objects (Table 1).

To bridge this gap, this work adopts a *safety-centric lens* to review and analyze state-of-the-art methodologies in DOM. Our approach is inspired by biological systems, where safety in the manipulation of fragile objects is achieved through synergistic, multi-level sensorimotor strategies. Fast reflexive responses safeguard against immediate risks, while cognitive planning and predictive modeling ensure long-term adaptability and task-level safety [17]. These systems rely on the integration of proprioception, tactile sensing, and adaptive modeling and control mechanisms [18,19,20,21]. However, current robotic systems often lack these critical capabilities—struggling to achieve the balance between rapid responsiveness and context-aware adaptability required for fragile object manipulation [22]. By systematically analyzing existing research and introducing a safety-oriented perspective, this review seeks to unify efforts toward safer, more adaptive frameworks for DOM. We aim to inspire transformative advancements that will broaden the adoption of autonomous robots in both domestic and industrial settings while enhancing their reliability in delicate and safety-critical applications.

Adopting this bio-inspired perspective, this review aims to

1. Systematically review the state of the art in DOM by analyzing the foundational roles of **hardware morphology**, **sensing**, **modeling**, **and control** in the context of fragility.
2. Structure this analysis using a **hierarchical framework** that distinguishes between slow, deliberative **cognitive control** and fast, **reflexive safety mechanisms**.
3. Argue that **proprioception** is the critical, synergistic bridge between these control layers and propose it as a key direction for creating more robust and adaptive systems.

By systematically analyzing existing research and introducing a safety-oriented perspective, this review seeks to unify efforts toward safer, more adaptive frameworks for DOM. We aim to inspire transformative advancements that will broaden the adoption of autonomous robots in both domestic and industrial settings while enhancing their reliability in delicate and safety-critical applications.

## 2. The Role of Hardware in Fragile Object Manipulation: Gripper Morphology

The physical design of an end effector is a foundational element in any manipulation task, establishing a baseline of passive safety that directly impacts the complexity of the required control and sensing frameworks. A comprehensive review of all robotic gripper hardware is beyond the scope of this paper. Instead, this section compares the two primary archetypes—rigid and soft grippers—to analyze their inherent advantages and limitations in the context of handling deformable and fragile objects.

### 2.1. Soft Grippers

Soft robotic grippers are designed with inherent compliance [23], typically using materials like silicone rubbers or thermoplastic polyurethane (TPU) [24]. Their core principle is to achieve safe and adaptive grasping through morphological computation, where the physical structure and material properties of the gripper perform part of the control task passively [25]. The primary advantages of soft grippers are their ability to conform to an object’s shape, distribute contact forces evenly, and absorb collision impacts, making them intrinsically safer for interacting with delicate objects [26].

The field of soft grippers is diverse, encompassing a range of actuation and adhesion principles [27]. To structure our discussion, we will focus on the key technologies identified in the literature, particularly for agricultural applications where handling fragile items is common. These technologies include the following:

- **Fluidic Elastomer Actuators (FEAs):** This is one of the most common technologies; they utilize chambers within a soft body that are actuated by positive (pneumatic) or negative (vacuum) pressure to create bending or other motions [28].
- **Tendon-Driven Passive Structures:** These grippers combine compliant, passive fingers with external actuators (like motors) that pull on tendons or cables to close the grip. This approach allows for soft contact while leveraging conventional actuation [29].
- **Granular and Particle Jamming:** These grippers consist of a membrane filled with granular materials. In its loose state, the gripper can conform to an object; when a vacuum is applied, the particles jam together, creating a solid grip that is highly adaptive to irregular shapes [30].
- **Controlled Adhesion Mechanisms:** Rather than applying force, these grippers use principles like electro-adhesion (electrostatic fields) or gecko-adhesion (van der Waals forces) to lift objects with an extremely light touch, making them ideal for the most delicate items [31].

### 2.2. Rigid Grippers

Rigid grippers represent the conventional standard in robotics, and they are typically constructed from hard materials like metal or plastic. These grippers consist of one or more pairs of non-compliant fingers actuated to precise positions. Their primary advantages are high precision, strength, and durability. The kinematics of rigid grippers is well-defined, simplifying the modeling and control for predictable interactions with known objects. However, these same properties present significant challenges when manipulating fragile or deformable objects. The fundamental limitation of a rigid gripper is its lack of passive compliance, which leads to two key problems for fragile object safety:

- **Stress Concentration:** Contact is often limited to a few points or lines, which can create high pressures that bruise, fracture, or otherwise damage delicate structures.
- **Sensitivity to Errors:** Small uncertainties in object position, shape, or the control model can lead to the application of excessive force, as the gripper cannot physically yield. The interaction is inherently less robust and safe for the object compared to a soft gripper.

Consequently, the burden of ensuring a safe grasp with a rigid gripper shifts entirely from the mechanical design to the computational intelligence of the system. Safe manipulation becomes dependent on a sophisticated, closed-loop framework of real-time sensing and adaptive control to precisely regulate interaction forces. This is often the only viable approach in applications like robotic surgery or certain food processing lines, where requirements for sanitation, cost, and reliability favor the use of rigid end effectors.

### 2.3. Comparison and Trade-Offs

The choice between a rigid and a soft gripper represents a fundamental trade-off between precision and passive safety. While soft grippers excel in unstructured environments by offloading control complexity to their mechanical design, rigid grippers remain the standard in many industrial and medical applications where strength, precision, and reliability are paramount. The optimal choice is not universal but depends on the specific object, task, and operational constraints.

Table 2 summarizes the primary trade-offs between these two design philosophies in the context of fragile object manipulation.

Ultimately, regardless of the hardware choice, the system’s ability to safely handle a fragile object depends on an intelligent computational framework. A “smart” controller can lend a soft touch to a rigid hand, while a soft gripper’s effectiveness is amplified by robust perception and planning. The following sections will therefore delve into the state-of-the-art models in sensing, modeling, planning, and control that enable this crucial intelligence.

## 3. Sensing and Perception

**Figure 1:**
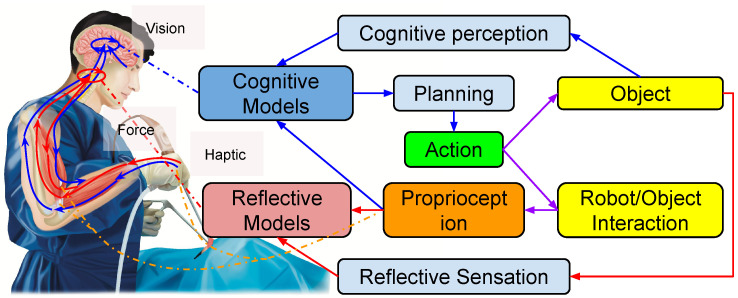
Conceptual illustration of the proposed hierarchical framework for fragile object manipulation using the analogy of a human surgeon. A surgeon seamlessly integrates multi-modal sensory information to handle delicate tissues safely: (a) Vision from an endoscope provides the global context, informing the slow, deliberative cognitive loop (e.g., planning a dissection path). (b) Haptic feedback felt through the surgical tools provides real-time data on contact forces, informing the fast reflexive loop (e.g., reacting to unexpected tissue resistance). (c) Proprioception—the surgeon’s innate sense of their own hand position and effort—acts as the crucial synergistic bridge. It grounds the cognitive plan in physical reality and enables the instantaneous reflexes required for safety.

Sensing and perception are fundamental to robotics and have been comprehensively reviewed [32,33,34]. Being different with other works, this work focuses on the specific and often unmet challenges that perception presents in the context of deformable object manipulation (DOM). In DOM, the core perceptual challenge is to provide the necessary information for a robot to infer an object’s complex shape, material constraints, and state during interactions. These modalities provide the essential data streams that inform the different layers of a hierarchical control system, from slow, deliberative planning to fast, reflexive actions. A schematic overview of how these sensors are integrated is shown in Figure 1.

### 3.1. Vision Sensing and Perception

Vision-based methods are the predominant approach in perception, and they are valued for their ability to extract global information about an object’s shape and motion. Depending on the application, a variety of imaging technologies are employed to capture object geometry and deformation. These include monocular cameras [35], stereo vision systems [36], and RGB-D cameras that provide direct depth measurements [37]. For high-speed dynamic scenarios, event cameras have also been utilized to track rapid changes [38].

While vision systems excel in geometric reconstruction, they face limitations when interacting with fragile objects [17]. Vision is often insufficient for detecting internal stress distribution or micro-scale deformations, which are critical in fragile contexts [39]. Furthermore, visual occlusion during manipulation, irregular surfaces, or transparent materials (e.g., glass) can hinder performance [40]. Stress prediction based solely on vision is unreliable and can result in oversights during manipulation, leading to damage [41]. As such, vision serves as the primary input for the **cognitive control loop**, providing the global context necessary for high-level task planning.

### 3.2. Tactile Sensing and Perception

Tactile sensors serve as the primary modality for acquiring rich, local information through direct physical contact [42]. Unlike remote sensors such as cameras, touch provides high-fidelity data about the interaction between the manipulator and an object’s surface [43]. This information is crucial for DOM, especially when manipulating fragile objects, as it enables the real-time control of forces and the detection of critical events like slip, which are often invisible to vision.

The data provided by tactile sensors can be understood in a hierarchy. At the most fundamental *contact level*, these sensors measure parameters like normal and shear forces, the position of contact, and local surface geometry [44]. This raw data can be processed to infer *object-level* properties, such as texture, compliance, or thermal characteristics. At the highest *action level*, this information is used to guide manipulation, for example, by adjusting grip force in response to incipient slips detected through vibrations or by confirming a stable grasp has been achieved.

While a wide variety of tactile sensing technologies exist, a prominent recent trend is the development of vision-based tactile sensors, such as GelSight or TacTip [45,46]. These sensors typically use an internal camera to observe the deformation of a soft, often marker-patterned skin. This approach provides a high-resolution “tactile image” from which detailed 3D shapes, textures, and force distributions can be reconstructed with remarkable precision. For fragile objects, such sensors are particularly beneficial as they can detect subtle force thresholds and surface changes that are critical for preventing damage.

Despite these advancements, tactile sensing in robotics still faces challenges. A key limitation is the gap between the predominantly reactive nature of current robotic systems and the active perception employed by humans, where exploratory actions are proactively used to gather tactile information [42,47]. Integrating this active-sensing paradigm remains a significant frontier for making robotic manipulation more intelligent and adaptive [48].

### 3.3. Force/Torque Sensing and Perception

While tactile sensors excel at providing high-resolution local contact information, force/torque (F/T) sensors offer a complementary, global perspective on the physical interaction between the manipulator and an object [49]. Typically mounted at the robot’s wrist, an F/T sensor measures the net forces and torques resulting from the entire interaction, providing a direct, physically interpretable measure of the overall load on the end effector [49,50]. This modality is crucial for tasks requiring precise force control, such as assembly, insertion, or carefully handling fragile objects where the total applied force must be kept below a critical threshold to prevent damage.

In the context of deformable object manipulation (DOM), F/T sensing provides vital feedback for executing contact-rich tasks. For fragile objects, it enables robots to limit exerted forces below breaking or permanent deformation thresholds. However, a primary limitation of F/T sensing is its lack of spatial resolution [51]. Because it measures the aggregate load, it cannot distinguish between different contact points or provide information about the pressure distribution across a surface. This ambiguity can make it challenging to diagnose the cause of unexpected forces, especially in multi-contact scenarios. Therefore, F/T sensing is most powerful when fused with other modalities, such as vision or tactile feedback, as it combines global dynamic information with the local geometric context.

Therefore, F/T sensing is most powerful when fused with other modalities, such as vision or tactile feedback, as it combines global dynamic information with the local geometric context (see Table 3).

### 3.4. Challenges in Fragile Object Sensing and Perception

Current sensing modalities face several challenges when applied to fragile object manipulation:

- **Stress and Strain Detection**: Vision systems struggle with detecting internal stresses, while tactile sensors are limited to surface interactions, leaving blind spots in real-time fragility assessments.
- **Occlusion and Transparency**: Vision sensors fail in occluded environments or with transparent objects, negatively impacting safe manipulation tasks.
- **Bandwidth Limitations**: High-bandwidth tactile feedback required for fragile object handling introduces complexities in both data acquisition and processing speeds.
- **Sensor Fusion**: Effective integration of multiple sensing modalities (vision, tactile, and force/torque) remains a challenge, particularly in fragility-aware systems requiring fine-grained real-time feedback.

### 3.5. Opportunities

To enhance perception for fragile object manipulation, future research must focus not only on improving individual sensors but also on advancing key technologies and integrating them into cohesive systems. The most prominent opportunities include the following:

- **Vision-Inferred Tactile Sensing:** Beyond dedicated hardware like GelSight (which uses an internal camera), a prominent research direction uses *external* vision to infer tactile properties. By observing an object’s deformation, these methods can estimate contact forces and pressures without direct contact, offering a powerful solution for environments where physical tactile sensors are impractical or infeasible [52].
- **Leverage Proprioceptive Force Estimation:** Using the robot’s own dynamic model and motor currents to estimate contact forces offers a low-cost, universally applicable alternative to dedicated sensors [53]. Future work must focus on creating highly accurate models and robust filtering techniques to disentangle delicate contact forces from the robot’s own dynamic noise.
- **Advance Holistic Sensor Fusion:** The future of perception lies in methodologies that intelligently fuse the global context from vision with high-frequency local data from tactile sensors and the global interaction dynamics captured by force/torque feedback (either measured or estimated).

## 4. Modeling Deformable and Fragile Objects

A foundational step in deformable object manipulation (DOM) is choosing an object model that balances representational fidelity with computational speed—a trade-off that is especially critical when handling fragile objects. Successfully modeling a deformable object requires addressing two distinct but related challenges: first, selecting a *representation* for the object’s geometry and second, applying a *model* to predict its physical behavior under applied forces. The following sections discuss these challenges in turn.

### 4.1. Model Representation

Model representation is a challenging and widely studied problem [54]. In DOM, the choice of geometric representation dictates how an object’s shape is discretized and tracked. This choice is fundamental, as it impacts the performance and complexity of any subsequent physical model. Common representations range from coarse meshes, which are computationally efficient and allow for real-time collision checks [55], to denser point-cloud [56] or signed distance field (SDF) [57] representations that enable more precise tracking of complex deformations. Each representation offers a different balance between geometric fidelity and the computational cost of updating it over time. The trade-offs for these common methods are summarized in Table 4.

### 4.2. Analytical Models

Analytical models predict object deformation by applying the first principles of physics to a chosen geometric representation. These models are often favored when physical accuracy is paramount, but they present a significant trade-off between fidelity and computational speed [10,58].

The most accurate and widely used analytical approach is the **Finite Element Method (FEM)**. By discretizing an object into a mesh of finite elements, the FEM can solve complex continuum mechanics equations to compute internal stress and strain distributions with high precision. This capability is invaluable for fragile object manipulation, as it allows for the prediction of potential failure points. However, the high computational cost of the FEM is a major barrier, often making it too slow for the real-time feedback required in robotic control loops [58].

To address the speed limitations of the FEM, discrete models like **mass–spring models (MSMs)** and, more recently, **position-based dynamics (PBD)** are commonly used. These methods offer much faster simulation speeds, making them suitable for interactive applications. Their limitation, however, lies in their physical realism. The parameters of an MSM, for example, often do not correspond directly to real-world material properties, leading to simulations that may look plausible but are not physically accurate. This lack of guaranteed fidelity poses a significant risk when manipulating fragile objects, where misjudging material responses can lead to damage.

Ultimately, the choice of an analytical model is dictated by this fundamental trade-off. For tasks involving fragile objects, there is a critical need for models that can bridge the gap between the accuracy of the FEM and the real-time performance of simpler discrete methods [58]. This trade-off maps directly onto a hierarchical control architecture: high-fidelity models like the FEM are suitable for offline simulations and the deliberative cognitive loop, while faster models like PBD are necessary for the real-time predictions required by the reflexive loop.

### 4.3. Data-Driven Models

Methods using **implicit neural representations** (such as NeRFs), **deep signed distance functions (SDFs)**, and **diffusion models** can learn to generate high-fidelity 3D models from a collection of 2D images [59,60,61]. For DOM, these techniques are promising because they can reconstruct the complex, non-rigid topology of a deformable object, even from partial or incomplete views. However, a primary challenge is that most of these methods are computationally intensive and optimized for static scenes [54]. Adapting them for the real-time tracking of dynamically deforming objects is a significant and active area of research.

Local tactile and force measurements can be used for object modeling through a process of interactive exploration and data fusion [47]. This process involves the robot interactively probing the object at multiple locations to gather local data, thereby addressing the ambiguity of data alignment [62]. At each point of contact, tactile sensors can measure high-resolution data like pressure distribution, while force sensors measure the interaction force required to cause a certain deformation. This local force–deformation data is then used to infer an object-level property, such as the material’s stiffness or compliance, at that specific location.

### 4.4. Challenges in Fragile Object Modeling

Modeling approaches currently lack comprehensive mechanisms to handle fragility constraints effectively:

- **Stress Threshold Prediction**: Fragile objects require precise stress and deformation predictions to avoid local damage, which remains challenging for both analytical and data-driven models.
- **Dynamic Fragility Modeling**: Objects often change fragility conditions during manipulation (e.g., brittle transitions in glass or softening in tissues). Neither modeling approach fully accounts for these dynamic states.
- **Computational Trade-Offs**: Analytical models are computationally expensive for high-resolution fragility simulations, whereas data-driven approaches struggle with real-time safety guarantees.

### 4.5. Opportunities

Future research must integrate fragility-specific constraints into modeling frameworks, prioritizing safe predictions without sacrificing adaptability. This can be achieved by

- Developing hybrid models that incorporate analytical accuracy with data-driven flexibility to adapt to unforeseen fragility changes.
- Implementing real-time fragility monitoring through feedback loops, leveraging high-bandwidth proprioceptive sensing.
- Addressing computational challenges by optimizing algorithms for fragile object dynamics simulations without compromising safety.

By advancing modeling approaches to account for fragility, DOM systems can become both safer and more reliable, enabling applications in critical fields such as healthcare and delicate manufacturing.

## 5. Motion Planning for Deformable Object Manipulation

Motion planning for deformable object manipulation (DOM) is a formidable task due to the unique challenges posed by non-rigid materials. Unlike rigid-body planning, DOM planners must contend with infinite degrees of freedom, complex and often unpredictable nonlinear dynamics, and the constant possibility of self-collision [63]. Furthermore, any viable solution must handle uncertainty and operate within the real-time constraints of a robotic system [64]. In the context of a hierarchical control system, motion planning constitutes the cognitive loop, which is responsible for generating a high-level, goal-oriented strategy that can be executed and refined by faster, reactive controllers.

The strategies developed to address these challenges can be broadly categorized into two primary philosophies: those that rely on an explicit physics model and those that learn a manipulation policy directly from data. Many modern solutions also create hybrids of the two or use specialized representations and reactive control strategies to simplify the problem.

### 5.1. Model-Based Planning

Model-based approaches leverage a predictive model of the object, typically based on Finite Element Methods (FEMs) or mass–spring models (MSMs), to simulate how it will deform [65,66,67]. This simulated behavior is then integrated into a planning framework. Common strategies include using sampling-based planners (e.g., RRT), where each sample is validated through simulations, or employing optimization-based planners (e.g., trajectory optimization) that incorporate soft-body physics as constraints [68]. While these methods can be highly physically realistic, their effectiveness is entirely dependent on the accuracy of the underlying model, and they are often too computationally expensive for real-time control [69].

### 5.2. Learning-Based Planning

Learning-based approaches bypass the need for an explicit analytical model by learning a control policy from data. This is typically achieved through

- **Imitation Learning (IL),** where a policy is learned from expert demonstrations [70].
- **Reinforcement Learning (RL),** where a policy is learned through trial and error to maximize a reward signal [71].

These methods excel at learning complex policies that can adapt to real-world noise and sensory feedback (e.g., from vision or touch). However, they typically require vast amounts of data to train and may struggle to generalize to novel situations or provide formal safety guarantees.

### 5.3. Feedback-Based Control and Visual Servoing

Distinct from deliberative planners, feedback-based strategies use continuous sensory input to guide the robot’s motion in real-time based on known policies [72]. A prime example is **visual servoing**, where features in an image are used to derive control signals that drive the robot, closing the loop through the camera [39]. This approach is highly reactive and less dependent on an accurate predictive model. Its primary drawback, however, is a high sensitivity to perception errors and occlusions, which are common in DOM tasks.

### 5.4. Challenges in Planning for Fragile Objects

Planning systems for fragile objects face several fundamental challenges:

- **Integration of Fragility Constraints**: Existing planning methods rarely embed fragility-related thresholds, such as limits on stress, strain, or applied force, into the cognitive loop’s trajectory generation.
- **Adaptiveness to Uncertainty**: Analytical and heuristic methods struggle to adapt when sensory feedback suggests dynamic changes in object fragility during manipulation.
- **Real-Time Decision-Making**: The computational cost of planning makes it difficult for the cognitive loop to react to fast, unexpected events, placing a heavy burden on lower-level reactive controllers.
- **Task-Specific Limitations**: Many planning frameworks are designed for specific applications (e.g., garment handling and food preparation) and are not generalizable to objects with diverse fragility profiles.

### 5.5. Opportunities

To improve planning for fragile object manipulation, future research must address the outlined limitations by focusing on the following:

- **Fragility-Aware Planning Models**: They develop planning frameworks for the cognitive loop that incorporate safety constraints directly into trajectory generation, using fragility predictions derived from sensing.
- **Hybrid Planning Architectures**: They combine the deliberative efficiency of the cognitive loop with the rapid error correction of the reflexive loop while embedding fragility rules to achieve both safety and flexibility.
- **Bio-Inspired Predictive Planning**: It takes inspiration from biological cognitive systems that integrate proprioception, vision, and tactile feedback for predictive adjustments during manipulation.
- **Real-Time Planning Optimization**: It enhances computational efficiency for learning-based approaches to enable real-time fragility-aware decision-making.
- **Multi-Object Planning Integration**: It expands existing frameworks to handle interactive tasks involving multiple fragile objects, such as simultaneous handling or assembly.

By advancing planning frameworks to account for fragility-specific constraints and safety considerations, DOM systems can achieve optimized trajectories that balance task success and damage prevention. This transformation is crucial for applications requiring safety-critical manipulation tasks, such as surgery, food handling, and glass manufacturing.

## 6. Control for Deformable Object Manipulation

While planning determines a high-level strategy, the control system is responsible for executing that strategy and making real-time adjustments to safely interact with the object. For deformable object manipulation (DOM), control is especially critical for managing contact forces and reacting to unexpected deformations. The primary control strategies can be categorized by their reliance on a physical model, their use of direct sensor feedback, or their foundation in machine learning.

### 6.1. Model-Based Control

Model-based control strategies leverage a known or approximated physical model of the object’s deformation to derive control laws.

**Model Predictive Control (MPC):** This advanced technique uses a predictive model (e.g., based on the FEM or the mass–spring model) to forecast the object’s future states [65,66,73]. At each time step, it calculates an optimal sequence of control inputs to follow a reference trajectory or achieve a desired deformation. While MPC is powerful due to its predictive and optimal nature, its effectiveness depends entirely on the accuracy of the underlying model, and its significant computational expense can be prohibitive for real-time applications [74].

#### Impedance and Admittance Control

These methods control the interaction by regulating the relationship between force and motion [75]. Instead of commanding a strict trajectory, the robot behaves as a programmable spring–damper system [76]. This approach is excellent for ensuring safe physical interactions by making the robot compliant. However, it offers less direct control over the object’s specific deformation, focusing more on the interaction forces than the resulting shape.

### 6.2. Model-Free Feedback-Based Control

In contrast to model-based methods, feedback-based control avoids explicit predictive models and instead relies on continuous, real-time sensor data to correct the robot’s motion [21].

#### 6.2.1. Visual Servoing

As a real-time feedback control strategy, visual servoing uses features from an image stream to derive motor commands. The goal is typically to drive the robot’s motion to make the current image match a target reference image. While highly reactive and not dependent on a physics model, visual servoing is very sensitive to camera calibration, perception errors, and especially occlusions, which are common in manipulation tasks [77].

#### 6.2.2. Tactile Feedback Control

This approach closes the control loop using data from tactile or force sensors [20]. By directly measuring contact information, the robot can adjust its grip force or pose to maintain a stable grasp or gently manipulate a surface. This method offers high sensitivity to contact events but is inherently limited by the spatial coverage of the sensor array.

### 6.3. Learning-Based Control

The dominant modern paradigm is to learn control policies directly from interaction data, bypassing the need for hand-crafted models or control laws.

**Reinforcement Learning (RL):** RL enables a robot to learn an optimal control policy through trial and error by maximizing a reward signal. It is extremely flexible and can learn to solve highly complex tasks. Its main drawbacks are the need for very large amounts of training data (often gathered in simulations, leading to a “sim-to-real” gap) and challenges in ensuring safety during the learning process.

**Imitation Learning (IL):** Also known as behavior cloning, IL learns a control policy by mimicking expert demonstrations. This approach is far more sample-efficient and safer to train than RL. However, the resulting policy is fundamentally limited by the quality of the demonstrations and may fail to generalize to states not seen during training. These learned policies are often implemented as neural feedback systems that map sensor observations directly to control commands in real time.

### 6.4. Challenges in Control for Fragile Objects

The reviewed control strategies face numerous challenges when considering the safety of fragile objects:

- **Lack of Fragility Constraints:** Control strategies, especially in Reinforcement Learning, often optimize for task completion without explicit fragility-aware parameters. Reward functions may not sufficiently penalize actions that cause subtle damage, and policies learned via Imitation Learning can fail when encountering unseen states where the object’s fragility becomes a factor.
- **Computational Bottlenecks:** Model-based controllers like MPC, while capable of predictive planning, often cannot meet the real-time computational requirements for safety-critical tasks. The delay in optimizing a new plan can be longer than the time it takes to irreversibly damage a fragile object.
- **Response Latency and Sensor Limitations:** The effectiveness of any feedback-based control is limited by sensor and processing latency. For fragile objects, even a small delay in detecting a force spike or slip from visual or tactile data can be the difference between a successful manipulation and a failed one. Furthermore, the limited spatial coverage of tactile sensors means that the controller is blind to damaging events happening outside the contact patch.
- **Generalization Gaps:** Learning-based methods frequently fail to generalize from simulations to the real world or from training objects to new ones with different fragility properties. A policy trained to handle a firm object may apply excessive force when confronted with a softer, more delicate variant.

### 6.5. Future Opportunities in Control

To develop fragility-aware control frameworks, future efforts should prioritize the following research directions:

- **Fragility-Aware Learning:** A significant opportunity lies in incorporating fragility constraints directly into the learning process. This can be achieved through safety-constrained reward functions, intrinsic penalties for high forces or rapid deformations, or by training a dedicated “safety critic” that evaluates the risk of an action in parallel with the main control policy.
- **Hybrid Control Systems:** Future work should explore hybrid frameworks that combine the predictive, optimal nature of model-based controllers with the rapid response of reactive mechanisms. For example, high-level MPC could plan a safe, long-horizon trajectory, while a low-level impedance controller or a simple reflexive loop provides an instantaneous safety net against unexpected forces.
- **Hierarchical and Bio-Inspired Control:** There is great potential in exploring hierarchical architectures that mimic biological systems. These would feature a high-level cognitive layer for strategic planning and a low-level reflexive layer that handles immediate safety based on high-frequency feedback from proprioceptive or tactile sensors, creating a system that is both intelligent and robustly safe.

## 7. Rethinking Fragile DOM: Key Challenges and a Path Forward

### 7.1. The Primary Challenge: Defining and Modeling Fragility

Manipulating fragile objects introduces unique challenges that require systems to consider not only the object’s deformability but also its susceptibility to physical damage, such as tearing, fracturing, or crushing. Fragility constraints relate to physical limits on stress, strain, or applied forces beyond which the object’s structural integrity is compromised. Existing approaches to DOM often address fragility as a secondary or ad hoc consideration, treating object safety as task-specific rather than embedding it as a core feature of the entire system.

This challenge is twofold, as fragile objects exhibit distinct characteristics that can be classified into *global fragility* and *local fragility*:

- **Global Fragility**: Some objects, such as glass rods or thin sheets, exhibit fragility thresholds determined by cumulative stresses from all interactions. Existing approaches that estimate global stresses often focus on force/torque balance but rarely incorporate long-term fatigue or stress accumulation during extended manipulation tasks.
- **Local Fragility**: For objects like soft tissues or brittle composites, damage may result from localized forces concentrated at specific points of contact. Current tactile- and force/torque-sensing systems are limited in detecting and predicting these localized risks, especially without detailed internal stress models.

The lack of standardized models for integrating both global and local fragility constraints presents a critical gap in current DOM research. Any successful path forward must begin by addressing this foundational challenge.

### 7.2. The Need for Predictive and Adaptive Models

For tasks involving fragile objects with immense variability, such as soft tissues, real-time adaptation is not just a feature but a necessity. The constant potential for unexpected events means that purely model-free or reactive methods, which rely on extensive trial and error, are often insufficient, as discussed in Table 5. To operate safely, a system requires an internal physical model that allows it to predict the consequences of its actions. This enables the robot to anticipate and avoid harm proactively rather than just reacting to damage after it has occurred.

An internal model is only effective if it accurately reflects the current state of the environment. To maintain this accuracy, the system must continuously learn and adapt its model parameters on the fly using sensory feedback. Vision, haptics, and force sensing are critical modalities for perceiving object properties such as tissue stiffness and deformation [43]. However, in constrained environments like surgical settings, the use of physical force or tactile sensors is often impractical due to challenges related to sterilization, miniaturization, and cost [97]. This limitation has led to the development of virtual sensors, such as a visual force proxy, which estimate interaction forces using vision and other data streams in lieu of dedicated physical sensors.

### 7.3. The Disconnect Between Deliberative Planning and Real-Time Control

A third major challenge lies in the frequent disconnect between long-horizon planning and real-time control. In theory, these systems should work together seamlessly; in practice, they often operate as distinct layers with limited communication, creating a gap that is particularly dangerous for fragile objects.

On one hand, cognitive-level planning relies on higher-level architectures that integrate multi-modal sensory data and internal models to anticipate risks and optimize manipulation strategies over longer time horizons. These systems are essential for complex tasks requiring foresight, such as surgical robotics or multi-step assembly. Their primary limitation, however, is computational inefficiency; they are often too slow to adapt in real time if a fragile object’s properties change or an unexpected event occurs.

On the other hand, low-latency control offers immediate, reflexive corrections based on high-frequency feedback from proprioceptive, force, and tactile sensors. These systems are effective for tasks like correcting a slip or modulating grip force. Their limitation is a lack of foresight; they can react to a problem, but they cannot anticipate and plan to avoid it. The core challenge is that most current systems lack a robust, synergistic bridge between these two essential layers.

### 7.4. A Path Forward: Close the Perception, Modeling, Planning, and Control Loop via Proprioception

The challenges of modeling fragility, maintaining adaptive internal models, and bridging the gap between slow planning and fast control all point toward the need for a more integrated and unified framework. A promising path forward lies in a bio-inspired, hierarchical architecture that does not merely layer these components but unifies them through a single, foundational sensory modality.

The key to unifying these components is proprioception [18]. Defined as the perception of the robot’s own kinematic and dynamic state (position, velocity, and force), it is the sensory modality that connects all other observations. Proprioception, or a subset thereof, has been applied in robotics to improve adaptability to dynamic environments, as summarized in Table 6.

While vision provides static snapshots of the scene, proprioception continuously and directly reflects the physical state changes that result from tool–object interactions [108]. More importantly, it serves as the causal link between robot action and physical consequence.

By elevating proprioception from a simple state-sensing tool to the central, synergistic bridge of the control architecture, the cognitive–reflexive gap can be closed. This principle is analogous to a human surgeon, who intuitively fuses multi-modal sensory feedback—vision, haptics, and proprioception—to continuously update their strategy and ensure safety during a delicate operation (Figure 1).

Its high-bandwidth feedback can drive the fastest, lowest-latency safety reflexes while simultaneously providing the ground-truth data needed to update the cognitive layer’s predictive models in real time. This proprioceptive feedback, which directly reflects the physical consequences of the robot’s actions, provides the ideal data stream for causal learning models to understand the dynamics of fragile object interaction, making the framework both adaptive and robust [109].

## 8. Conclusions

Deformable object manipulation (DOM) represents one of the most complex challenges in robotics, particularly when handling fragile objects. Ensuring safety requires integrating fragility constraints at every level of system design, from hardware to control. This review has provided a safety-centric analysis of the field, organizing the state-of-the-art studies within a bio-inspired, hierarchical framework to identify critical gaps and propose a path forward.

Through this analysis, several key insights have emerged:

- The choice of **hardware morphology**, from rigid to soft grippers, establishes a baseline of passive safety that fundamentally shapes the subsequent control challenges.
- A primary limitation in current systems is the **cognitive–reflexive gap**—a disconnect between slow, deliberative planners and fast, reactive controllers that hinders real-time adaptation.
- External sensing modalities like vision and touch, while critical, are often insufficient without being grounded by high-bandwidth **proprioception**, which provides the direct causal link between action and consequence.

Based on these insights, we argue that the path forward lies in developing unified, hierarchical frameworks that bridge the cognitive–reflexive gap. The key to this integration is elevating proprioception as the synergistic sensory bridge and leveraging it to power causal learning models. Such a system would be capable of both long-horizon planning and instantaneous, safe adaptation, addressing the specific constraints of diverse applications.

By shifting focus towards these integrated, proprioceptive-causal systems, the field can unlock transformative progress in critical domains like robotic surgery, agriculture, delicate manufacturing, and waste sorting. This review aims to unify efforts across hardware design, sensing, and control to enable the next generation of safe, robust, and adaptable robotic solutions. 

## Figures and Tables

**Table 1:** Summary of existing surveys on deformable object manipulation. This table contrasts prominent prior reviews by highlighting their primary focus areas and limitations. It demonstrates that while DOM has been reviewed broadly, a systematic analysis centered on the specific challenges of fragile object safety and integrated multi-modal sensing remains an underexplored area, establishing the novel contribution of this work.

Reference	Focus Area	Modalities	Noted Limitations
Gu et al. (2023) [9]	General review of DOM; data-driven and hybrid methods	Vision, tactile, and force	Limited mention of proprioception; minimal focus on fusion
Zhu et al. (2021) [10]	Challenges and future directions in DOM	Vision, force, and tactile	Suggests multi-modal fusion but without deep implementation details
Jiménez (2012) [11]	Model-based manipulation planning	Mostly modeling	Little discussion of sensing modalities
Herguedas et al. (2019) [12]	Multi-robot systems for DOM	Vision and force	Limited on tactile and proprioception; focuses on coordination
Arriola-Rios et al. (2020) [13]	Modeling of deformable objects for robotic manipulation	Vision and force	Focuses on object modeling; less discussion on action planning and multi-modal fusion
Yin et al. (2021) [1]	Modeling, learning, perception, and control methods	Vision and tactile	Briefly mentions force; lacks multi-modal integration
Kadi and Terzić (2023) [14]	Data-driven approaches for cloth-like deformables	Vision and tactile	Discusses challenges but does not cover proprioception deeply
Blanco-Mulero et al. (2024) [15]	Proposed taxonomy (T-DOM) for deformable manipulation tasks	Vision, force, and tactile	High-level categorization; not focused on sensing strategies
Sanchez et al. (2018) [16]	Robotic manipulation and sensing of deformable objects in domestic and industrial applications	Vision, force, and tactile	Broad classification across object types and tasks; limited depth on sensor-fusion strategies and minimal focus on proprioception

**Table 2:** Comparison of rigid and soft gripper archetypes for fragile object manipulation. This table highlights the fundamental trade-offs in performance, control, and practical application between the two primary hardware designs.

Feature	Rigid Grippers	Soft Grippers
**Passive Safety**	Low. Lacks compliance, creating a higher risk of damage from unexpected contact or control errors.	High. Inherently compliant materials absorb impacts and prevent force spikes, making interactions safer.
**Force Distribution**	Concentrated at a few points, leading to high stress that can easily damage fragile objects.	Distributed over a larger surface area as the gripper conforms to the object’s shape.
**Shape Adaptability**	Low. Requires a precise model of the object’s geometry for a successful grasp.	High. Can passively conform to a wide variety of regular and irregular shapes.
**Control Complexity**	High. Safety is highly dependent on sophisticated, real-time feedback and precise force control.	Lower. The gripper’s morphology handles much of the complexity, reducing the burden on the controller.
**Precision and Strength**	Typically high. Capable of precise positioning and applying significant force.	Generally lower. Precision can be more difficult to model and control; payload-to-weight ratios vary widely.
**Durability and Sanitation**	High. Often made of robust metals or plastics that are durable and easy to sterilize.	Varies. Soft materials can be susceptible to wear, tear, and contamination, posing challenges for some applications.

**Table 3:** A comparison of the primary external sensing modalities in deformable object manipulation (DOM). This table summarizes their inherent trade-offs, highlighting how the global, non-contact nature of vision complements the high-fidelity local data from tactile and force/torque sensors. The key takeaway is that each modality has significant limitations, such as occlusion in vision and limited coverage in touch, which underscore the need for multi-modal fusion.

Modality	Advantages of DOM	Limitations of DOM
**Vision**	• Global, non-contact sensing of shapes & motion	• Highly prone to occlusions Cannot measure contact forces or internal stress Poor with transparent or textureless objects
**Tactile**	• High-resolution local data (force, slip, and texture) High-frequency feedback for fine control	• Sensing area limited to direct contact Complex or costly to integrate large arrays
**Force/Torque**	• Measures net interaction force for global control Excellent for enforcing overall force limits	• Lacks spatial resolution (cannot localize contact) Sensitive to noise from the robot’s own dynamics

**Table 4:** This table summarizes common methods for representing deformable objects, highlighting the fundamental trade-off between approaches that offer high physical fidelity (e.g., FEM) and those that prioritize computational speed for real-time applications (e.g., mass–spring models). The choice of representation is a critical, task-dependent decision that directly impacts the performance of any subsequent planning or control system.

Method	Advantages	Disadvantages
Mesh-based	Real-time collision checks; straightforward to implement	Limited deformation fidelity; mesh artifacts under large strains
SDF	Smooth, continuous geometry; precise deformation recovery	High memory footprint; expensive distance queries
Mass–spring	Very fast simulation; intuitive parameter tuning	Oversimplified physics; cannot capture complex material behaviors
FEM	High-fidelity modeling; supports nonlinear constitutive laws	Computationally intensive; requires mesh generation and parameter tuning
Data-driven	Learns from real examples; often real-time inference	Data-hungry; limited interpretability; risk of overfitting and poor generalization

**Table 5:** This table classifies representative manipulation strategies according to the proposed hierarchical framework. Selected state-of-the-art works are mapped onto the three primary control loops: Spinal Reflex (<50 ms), Long-Latency Reflex (50–100 ms), and Cognitive (>100 ms). The comparison illustrates how different combinations of sensing and control are suited for either immediate, reactive safety or long-horizon, deliberative planning.

	Sensing Modalities	Control Method	Assigned Loop	Note
[78]	Joint torque	Ultra-fast proprioceptive collision detection within the joint servo driver	Spinal Reflex (<50 ms)	Leverages high-frequency torque error thresholds to instantly halt motor commands at sub-millisecond latencies without higher-level inference.
[79]	Joint torque	Hybrid variable admittance via Fuzzy Sarsalearning	Long-Latency Reflex (50–100 ms)	Adapts admittance gains online based on torque feedback, providing skill-tuned compliance in tens of milliseconds.
[80]	GelSight	Parallel PD grip control and LQR pose control on a learned linear model	Long-Latency Reflex (50–100 ms)	Runs lightweight learned models at ∼60–125 Hz on tactile cues to maintain cable alignment without full planning.
[81]	Proprioception, vision, and audio	HMM-based multimodal anomaly detection	Long-Latency Reflex (50–100 ms)	Fuses proprioceptive residuals with audio/vision in an HMM to quickly flag failures without deliberation.
[82]	RGB-D vision	Topological autoencoder + fixed-time sliding-mode controller (∼20 Hz)	Long-Latency Reflex (50–100 ms)	Provides reflexive shape corrections using low-dimensional latent models for real-time adaptation.
[83]	Wrist force/torque	Real-time elasticity estimation from force–position curves	Long-Latency Reflex (50–100 ms)	Infers material properties on the fly to adjust grasp strategies within tens of milliseconds.
[84]	Joint positions	Observer for force/velocity estimation + Bayesian parameter classifier	Long-Latency Reflex (50–100 ms)	Uses a state observer on proprioceptive data to infer forces and classify tissue parameters rapidly.
[85]	Joint encoder	Differentiable simulation pipeline for inverse parameter identification	Long-Latency Reflex (50–100 ms)	Inverts a differentiable model on high-rate encoder streams to infer mass and elasticity in real time.
[86]	Tactile	Slip detection via tangential-force thresholds + immediate position adjustment	Long-Latency Reflex (50–100 ms)	Detects slip through fast tactile thresholds and issues corrective motions to prevent object loss.
[87]	Vision, tactile, and encoder	HMM + kernel logistic regression + Bayesian networks	Cognitive (>100 ms)	Integrates multi-modal cues with probabilistic learning to predict and replan stable grasps.
[88]	Vision	Sequential RL for manipulation- primitive parameters	Cognitive (>100 ms)	Learns high-level parameter sequences for long-horizon cloth tasks via deliberative policy optimization.
[89]	Vision	RL with dynamic domain randomization (∼25 fps)	Cognitive (>100 ms)	Trains end-to-end visual policies for cloth folding through deliberative RL.
[90]	Vision, proprioception, and tactile	Predefined folding trajectories + sensory feedback	Cognitive (>100 ms)	Uses physics-based modeling and sensory fusion to plan multi-step folding sequences.
[91]	Joint torque	Supervised learning on haptic time series for classification	Cognitive (>100 ms)	Trains models on torque signatures to classify geometry/materials and inform high-level planning.
[92]	Force and proprioception	MPC with RNN/LSTM dynamics (∼10 Hz)	Cognitive (>100 ms)	Embeds learned RNN dynamics into MPC for deliberative adaptation to varied food properties.
[93]	Proprioception and dynamics	SVR on haptic histograms + Monte Carlo–greedy planning	Cognitive (>100 ms)	Builds latent haptic belief models to guide long-horizon manipulation planning.
[94]	IMUs	ConvBiLSTM regression on squeeze–release inertial data	Cognitive (>100 ms)	Learns inertial patterns to predict stiffness, informing subsequent manipulation trajectories.
[95]	Joint angles	Projected diagonal Kalman filters on spring--voxel models (∼23 Hz)	Cognitive (>100 ms)	Recursively updates voxel-wise stiffness estimates to support planning over object compliance.
[96]	RGB, F/T, joint encoder	Self-supervised latent fusion + deep RL	Cognitive (>100 ms)	Trains compact embeddings to improve sample-efficient, deliberative control in contact-rich scenarios.

**Table 6:** This table provides a survey of representative works that leverage proprioception for advanced object manipulation. It highlights the versatility of proprioceptive feedback by showing how its different components—**P**osition, **V**elocity, **T**orque, and **I**nertia—are integrated with external sensing modalities like **Ta**ctile and **Vi**sion. The diverse design philosophies demonstrate the potential of proprioception to serve as a synergistic foundation for multi-modal control.

	Proprioception				Ta	Vi	Design Philosophy
	**P**	**V**	**T**	**I**			
[98]	✓	✓	✓	–	✓	✓	Real-time fusion for deformable-object modeling and control
[99]	✓	–	✓	–	–	–	Proprioceptive torque/angle-based identification of flexible-loop spring constants via variational integrators.
[93]	✓	–	✓	–	–	–	Haptic (encoder + effort/F/T) fusion for deformable-food property estimation and planning (no velocity/IMU/tactile).
[100]	✓	–	✓	–	✓	–	Fusion of joint encoders and torque sensing with a tactile array for rigid vs. deformable classification (97.5% accuracy).
[94]	–	–	–	✓	–	–	Deep-learning stiffness regression using only IMU-based inertial proprioception (≤8.7 % MAPE).
[95]	✓	–	✓	–	–	✓	Real-time volumetric stiffness field estimation from joint torque and optional vision for heterogeneous deformables.
[85]	✓	–	–	–	–	–	Differentiable simulation for mass and elastic-modulus estimation from joint-encoder signals alone.
[101]	✓	–	–	–	–	–	Large-strain piezoresistive proprioceptive sensing for single-grasp object shape classification and curvature estimation.
[102]	✓	–	–	–	✓	✓	Neural-network–based vision–force fusion for predictive deformable-object modeling (no joint-torque/IMU proprioception).
[84]	✓	✓	–	–	–	–	Sensorless force/velocity estimation from joint positions and commanded torques for biomechanical parameter identification and classification in robotic palpation.
[83]	✓	–	✓	–	–	–	Online elasticity/viscoelasticity estimation from gripper position and F/T sensing for real-time material sorting.
[103]	–	✓	–	–	✓	–	Neuromorphic fusion for speed-invariant texture discrimination.
[104]	–	–	✓	–	✓	–	Learning soft-membrane dynamics from high-res tactile geometry and reaction wrenches for real-time dexterous control.
[105]	✓	✓	✓	–	–	–	TossNet: real-time trajectory prediction from end-effector poses, velocity, and F/T-based proprioception.
[106]	✓	–	–	–	✓	–	Four-dimensional ICP–based fusion of encoder positions and tactile codebook labels for high-accuracy shape recognition.
[107]	✓	–	✓	–	–	–	Bimanual in-hand object-pose disambiguation via iterative contact probing using only joint-encoder and wrist F/T feedback, which is refined by dual particle-filter estimation.
[91]	–	–	✓	–	–	–	Joint-torque-driven classification/regression for simultaneous estimation of object geometry and materials using kinesthetic sensing.
[96]	✓	✓	✓	–	–	✓	Variational self-supervised fusion of RGB-D, EE pose/velocity, and F/T for RL-based peg insertion (no IMU/tactile arrays).

## Data Availability

No new data were created or analyzed in this study.
